# Eugenol prevents amyloid formation of proteins and inhibits amyloid-induced hemolysis

**DOI:** 10.1038/srep40744

**Published:** 2017-02-01

**Authors:** Kriti Dubey, Bibin G. Anand, Dolat Singh Shekhawat, Karunakar Kar

**Affiliations:** 1 Department of Biology, Indian Institute of Technology Jodhpur, Rajasthan, 342011 India; 2 School of Life Sciences, Jawaharlal Nehru University, New Delhi-110067, India

## Abstract

Eugenol has attracted considerable attention because of its potential for many pharmaceutical applications including anti-inflammatory, anti-tumorigenic and anti-oxidant properties. Here, we have investigated the effect of eugenol on amyloid formation of selected globular proteins. We find that both spontaneous and seed-induced aggregation processes of insulin and serum albumin (BSA) are significantly suppressed in the presence of eugenol. Isothermal titration calorimetric data predict a single binding site for eugenol-insulin complex confirming the affinity of eugenol for native soluble insulin species. We also find that eugenol suppresses amyloid-induced hemolysis. Our findings reveal the inherent ability of eugenol to stabilize native proteins and to delay the conversion of protein species of native conformation into β-sheet assembled mature fibrils, which seems to be crucial for its inhibitory effect.

Eugenol (4-Allyl-2-methoxyphenol) is a phenolic natural compound which has gained a lot of attention in recent years because of its versatile pharmacological applications^1^ which include its anti-inflammatory, anti-tumorigenic and anti-oxidant properties^2^. Eugenol is mostly found in the essential oils extracted from clove, basil, cinnamon and bay leaf^3^,^4^. Eugenol is known to interact with different proteins as well as DNA molecules^5^,^6^,^7^ and to influence their functional properties. Antiasthmatic effect of eugenol has been recently reported in a mouse model where eugenol was shown to influence Vitamin D3 upregulated protein 1/NF-κB pathway^8^. Recently, the inhibition effect of eugenol on key enzymes related to diabetes and hypertension^2^ was reported in a study that involved both *in vitro* and *in vivo* model systems. Furthermore, it has been shown that eugenol suppresses the activity of Cl^−^ Channel TMEM16A^9^.

Neuroprotective nature of eugenol has been already reported using both *in vitro* and cellular model systems^10^,^11^. Since eugenol has the potential to interact with a wide range of proteins and reports on the effect of eugenol on amyloid formation of proteins are limited in literature, the question of what effect eugenol would have on the amyloid formation of proteins stands very significant. Some studies on cell models, however, have indicated that eugenol can protect PC12 cells from toxic amyloids^12^,^13^. Further, eugenol is also known to suppress the occurrence of dopamine depression and lipid peroxidation inductivity, which are directly linked to Parkinson’s disease^14^. Considering such neuroprotective nature of eugenol against toxic amyloids, elucidation of the effect of eugenol on amyloid formation of proteins becomes very important. The formation of amyloids is a fundamental process in biology and^15^,^16^ more than 35 different proteins are known to be associated with several amyloid-linked diseases^16^,^17^. Targeting the onset of amyloid formation is considered to be one of the critical strategies to prevent amyloid linked diseases and its associated medical severities.

Here, we demonstrate the inhibition of temperature induced amyloid formation of two selected globular proteins, insulin and serum albumin (BSA), in the presence of eugenol. The process of amyloid formation of both insulin and serum albumin is known to cause many complications^18^,^19^,^20^,^21^, and these globular proteins are also known to form amyloid fibrils under *in vitro* conditions^22^,^23^,^24^,^25^. The process of insulin aggregation is also a great concern for its storage as therapeutic agents^26^,^27^. Hence, we have selected these two proteins as convenient model systems to elucidate the anti-amyloid activity of eugenol. This study has mainly focused on two key issues. First, we have investigated eugenol’s inhibitory effect on both spontaneous and seed-induced amyloid formation of proteins, under *in vitro* conditions. Second, we have explored the protective effect of eugenol on amyloid-induced hemolysis of RBCs. Finally, on the basis of our results, we hypothesize that eugenol has an inherent ability to stabilize native conformations of proteins and to delay the formation of amyloid fibrils.

## Results

### Effect of eugenol on amyloid formation of proteins

**Figure 1:**
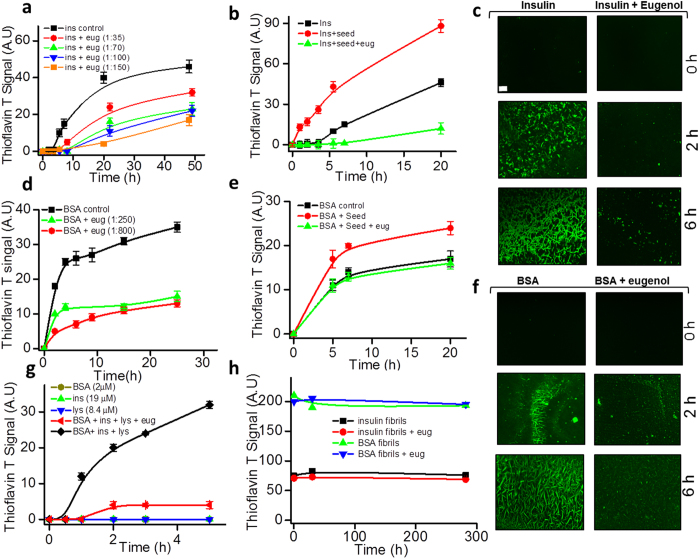
Inhibition of insulin and BSA amyloid formation in the presence of eugenol. (**a**) Effect of eugenol on spontaneous aggregation of insulin (~43 μM) at different molar ratios; 1:0 (■), 1:35 (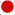), 1:70 (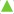), 1:100 (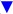) and 1: 150 (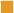). (**b**) Seed-induced aggregation of insulin (at ~43 μM): Insulin alone (■), insulin + seed (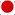); insulin + seed + eugenol at 1:100 molar ratio of protein:inhibitor (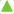). (**c**) Fluorescence microscopy images of Thioflavin T stained samples of insulin at different time points, as labeled. Scale bar, ~20 μm. (**d**) Effect of eugenol on spontaneous aggregation of BSA (at ~5 μM): BSA only (■); BSA + eugenol at 1:250 molar ratio of protein: inhibitor (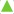); BSA + eugenol at 1:800 molar ratio of protein: inhibitor (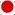). (**e**) Seed induced aggregation of BSA (~3 μM); BSA only (■), BSA + seeds (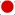), and BSA + seeds + eugenol (1:400 molar ratio) (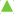). (**f**) Fluorescence microscopy images of Thioflavin T stained samples of BSA at different time points, as labeled. Scale bar, ~20 μm. (**g**) Effect of eugenol on coaggregation of BSA, insulin and lysozyme: ~2 μM BSA (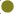), ~19 μM Insulin (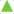), ~8.4 μM lysozyme (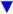), [2 μM BSA + 19 μM Insulin + 8.4 μM lysozyme] (♦), [2 μM BSA + 19 μM Insulin + 8.4 μM lysozyme + 3 mM eugenol] (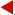). (**h**) Effect of eugenol on the disassembly process of matured amyloid fibrils of insulin and BSA; Insulin amyloids (■), insulin amyloids + eugenol (1:100 molar ratio of protein:inhibitor) (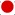), BSA amyloids (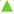), BSA amyloids + eugenol (1:400 molar ratio of protein:inhibitor) (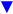). Until ~300 hr of observation, we did not see any indication of dissociation of amyloid fibrils. Seed implies 15% (w/w) preformed mature fibrils of the protein sample.

**Figure 2:**
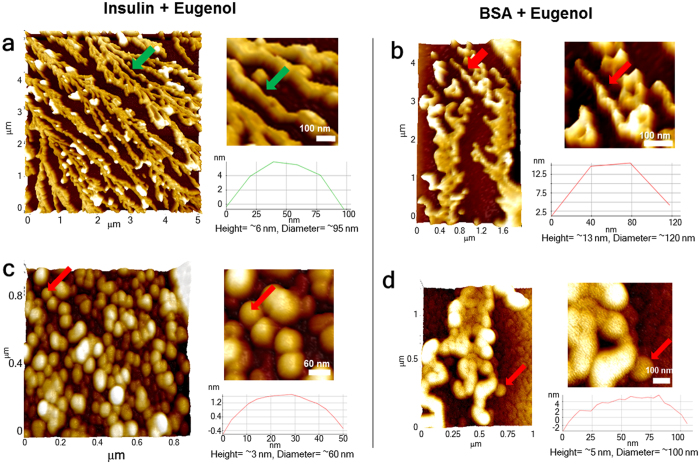
AFM images of aggregates of insulin and BSA obtained from eugenol-inhibited aggregation reactions. (**a**) AFM images of insulin mature fibrils obtained from an inhibited aggregation reaction which reveal a diameter value of ~95 nm (see inset). (**b**) Mature amyloid fibrils of BSA obtained from an inhibited aggregation reaction which show a diameter value of ~120 nm (see inset). (**c**) Occurrence of spheroidal oligomers of insulin with an average diameter of ~60 nm (see inset). (**d**) Morphology of oligomers or protofibrils of BSA shows an average diameter of ~100 nm. Mature aggregates of proteins in the absence of eugenol are shown in Supplementary Figures S2, and S3.

**Figure 3:**
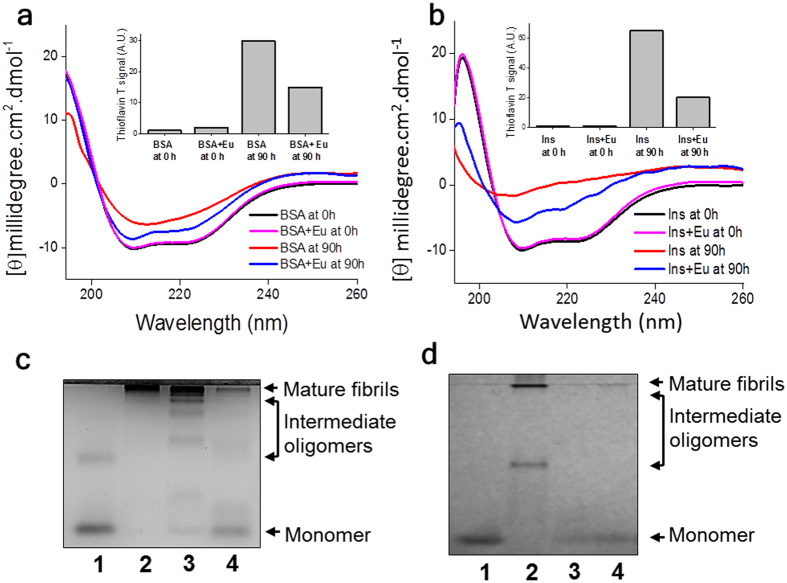
Structural studies on eugenol-protein interaction. (**a**) CD spectra of BSA undergoing aggregation in the presence and in the absence of eugenol (at molar ratio of 1:400 of protein:ligand): BSA at 0 h (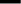); BSA + eugenol at 0 h (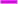); BSA at 90 h (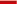); BSA + eugenol at 90 h (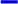). (**b**) CD spectra of insulin undergoing aggregation in the presence and absence of eugenol (at molar ratio of 1:100 of protein:ligand): insulin at 0 h (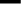); insulin + eugenol at 0 h (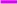); insulin at 90 h (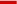); insulin + eugenol at 90 h (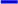). Insets of panel ***a*** and panel ***b*** show Thioflavin data for the respective CD samples. (**c**) Native gel-electrophoresis of the BSA (~5 μM) undergoing amyloid formation in the presence and in the absence of eugenol (at ~3 mM): (**1**) soluble BSA; (**2**) mature BSA aggregates; (**3**) BSA in the absence of eugenol at 5 h; (**4**) BSA in the presence of eugenol at ~5 h. (**d**) Native gel-electrophoresis of the insulin (~43 μM) undergoing amyloid formation in the presence and absence of eugenol at 1:150 molar ratio of insulin:inhibitor (**1**) soluble insulin; (**2**) mature insulin aggregates; (**3**) insulin in the absence of eugenol at ~6 h (**4**) insulin in the presence of eugenol at ~6 h. Uncropped native gel images are shown in Supplementary Figure S14.

Amyloid formation of both insulin and serum albumin was initiated by incubating the protein monomer samples in PBS (maintained at pH 7.4) at an elevated temperature close to their T_m_ values^25^ and the aggregation kinetics was monitored by measuring Thioflavin T readings of the samples at different time points. Formation of amyloid fibrils, as evident from the ThT signals (both for insulin and BSA), was observed to be substantially suppressed in the presence of eugenol (Fig. 1a,d). The protein samples undergoing amyloid formation were further examined at selected time points using fluorescence microscopy to capture the formation of ThT-sensitive amyloids as shown in Fig. 1c,f. The data as shown in panel c and panel f of Fig. 1 clearly support the kinetic data of the inhibitory effect of eugenol on amyloid formation of both insulin and BSA. To further confirm this inhibition effect, we then checked the effect of eugenol on fluorescence emission properties of ThT. Obtained data confirmed that eugenol itself does not alter the fluorescence properties of ThT (Supplementary Figure S9). Further, we did not see any such effect of ethanol on aggregation kinetics of proteins (Supplementary Figure S1). We also examined the effect of 2-methoxyphenol on the aggregation process of insulin and the obtained data (Supplementary Figure S12) did not show such strong inhibition effect like eugenol. Since the methoxyphenol moiety is a part of the eugenol structure, the data shown in Supplementary Figure S12 suggest that the inhibition effect may be specific to eugenol molecule. Hence, the decrease in ThT signals in our data signifies the inhibition of the amyloid formation in the presence of eugenol. The nature of the aggregation curves obtained from ThT assays indicated a decrease in the extent of amyloid formation in the presence of eugenol. We further have looked at AFM images of mature insulin fibrils (Fig. 2a) obtained from the inhibited aggregation reactions and the results showed typical amyloid morphology as seen for insulin^28^. However, we observed a large population of oligomeric species in the same sample (Fig. 2c). These spheroidal oligomers have also been observed to occur during the progression of protein amyloid formation such as in the case of insulin^29^ and α-synuclein^30^. We observed similar results when we looked at AFM images of samples taken from an inhibited aggregation reaction of BSA in the presence of eugenol (Fig. 2b). The oligomeric species of BSA displayed a proto-fibrillar appearance (as shown in Fig. 2d) rather than spheroidal structures as seen for insulin. Nevertheless, the mature fibrils taken from both inhibited and uninhibited reactions of proteins look almost similar for both insulin and BSA (Fig. 2 and Supplementary Figures S2 and S3). Considering the suppression of ThT signals and the occurrence of large population of oligomers in the inhibited aggregation reactions of proteins, we predict the ability of eugenol to retain the native protein species growing into mature fibrils. This interpretation is supported by our data obtained from native gel experiments (Fig. 3c,d).

In order to validate this interpretation, we studied the effect of eugenol on seed-induced aggregation of proteins. In the presence of ~15% (w/w) preformed fibrils (seeds), both insulin and BSA showed aggressive aggregation profiles (Fig. 1b,e). However, in the presence of eugenol, the seeded aggregation reactions of both insulin and BSA seemed to be suppressed (Fig. 1b,e). This suggests that the eugenol molecule is either capable of binding to the protein monomers or it may perhaps interact with the preformed amyloid fibrils or protofibrils, apparently blocking the growth phase of the aggregation reaction. We further examined the affinity of eugenol to bind to the amyloid fibrils by conducting sedimentation assays (see experimental section) where we observed a dose dependent affinity of eugenol molecule for mature amyloid fibrils of both BSA and insulin (Supplementary Figure S6). Though these results suggest ability of the eugenol molecule to interact with the fibrillar species of the protein, it was not clear whether the inhibitor molecules bind to the growing ends of the fibrils during aggregation.

### Effect of eugenol on coaggregation of proteins

Recently, under *in vitro* conditions, we reported the occurrence of rapid coaggregation of globular proteins into amyloid fibrils^25^ and in the same study the coaggregation process was found to have faster kinetics than the kinetics observed for individual aggregation reactions. We tested the effect of eugenol on the coaggregation process of three selected globular proteins (BSA, insulin and lysozyme). Figure 1e clearly indicates the process of rapid coaggregation (Fig. 1g,♦) of [BSA + insulin + lysozyme] sample because no aggregation was observed for individual protein samples (Fig. 1g,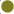 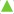 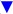). However, such coaggregation process was substantially suppressed in the presence of eugenol (Fig. 1g,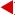). The ability of eugenol to inhibit both individual aggregation reactions and coaggregation reactions clearly suggests that the inhibition mechanism may involve common driving forces irrespective of the sequence identities of the aggregating species. To further our understanding of the eugenol-amyloid interaction, we have tested whether eugenol is capable of promoting disassembly of matured amyloid fibrils of proteins. Notably, we were unable to detect any effect of eugenol on the dissociation of amyloid fibrils (Fig. 1h).

The choice of at what concentration of the protein samples for aggregation experiments, as described above, was completely dependent on our previous knowledge, gained from the aggregation studies of BSA and insulin^25^,^31^. For studying coaggregation of proteins, we reduced the concentration of insulin from 43 μM to ~20 μM to substantially delay the lag time of individual aggregation process so that the occurrence of coaggregation can be detected clearly.

### CD-spectroscopy studies

To understand the structural changes during suppression of protein aggregation, we monitored CD spectra of the protein samples undergoing aggregation in the presence of eugenol at selected time points. We first looked at CD spectra of only eugenol sample (at the studied concentrations) in PBS buffer and the obtained data, as described in the Supplementary Figure S4, looked similar to the CD curves obtained for either water or PBS buffer. Hence, eugenol itself, at the studied concentrations, did not contribute to the ellipticity signals of CD data obtained for protein samples. Figure 3a,b compare the CD curves of both inhibited and uninhibited reactions of BSA (panel a) and insulin (panel b) recorded at 0 h and 90 h time points. The molecular conformations of BSA (Fig. 3a, black and magenta curves) and insulin (Fig. 3b, black and magenta curves) remain unchanged in the presence of eugenol at 0 h. However, during aggregation, it appears that the process of conversion of native protein molecules into β-sheet assembled species is delayed in the presence of eugenol (blue and red curves of Fig. 3a,b). We measured the ThT signals of the same CD samples and the data are shown as insets of Fig. 3a,b. ThT data strongly support our interpretation of CD results. Next, we performed native gel-electrophoresis experiments to gather further information on the ability of eugenol to retain native protein structures during suppression of protein aggregation process. The obtained data, as shown in Fig. 3c,d, clearly support this hypothesis.

### Intrinsic Fluorescence studies of eugenol-insulin interaction

Next, we used fluorescence technique to gain further information on insulin-eugenol interactions, particularly to understand the binding of eugenol with molecular insulin. Using established protocols^32^,^33^, we examined the quenching of eugenol in the presence of insulin and the obtained data were used to calculate Stern-Volmer constant *(K*_*sv*_), quenching rate constant (*K*_*q*_), binding constant *(K*_*a*_) and binding site (*n*) parameters. Eugenol emits fluorescence at 325 nm when it is excited at 262 nm^5^. The fluorescence spectra of eugenol in the presence of different concentrations of insulin are shown in Supplementary Figure S5a. The inset of Figure S5a shows the baseline corrected quenching data of eugenol in the presence of insulin. These data clearly reveal that fluorescence emission of eugenol was significantly quenched by insulin, and there was a gradual decrease in the eugenol fluorescence by increasing insulin concentration (inset Figure S5a). To calculate the magnitude and the nature of the quenching phenomenon in our study, the fluorescence emission spectra were analyzed using Stern-Volmer equation^34^,^35^ and the obtained plot is shown in Figure S5c. The values of *K*_*sv*_ and *k*_*q*_ were found to be 2.5 × 10^5^ L mol^−1^ and 2.5 × 10^13^ L mol^−1^ s^−1^ respectively. Since the value of *k*_*q*_ is greater than 2.0 × 10^10^ L mol^−1^ s^−1^, the quenching process of eugenol by insulin was predicted to be static. We further calculated the binding constant (*K*_*a*_) and binding site parameter (*n*) by using reported analysis equations^5^,^36^. The value of *K*_*a*_ was observed to be 5.8 × 10^3^ L mol^−1^ and the binding site parameter (*n*) was observed to be ~1.5, suggesting a single binding site.

### FTIR studies on protein aggregates

In order to examine the effect of eugenol on the structural properties of amyloid fibrils, we obtained FTIR spectra of BSA and insulin amyloid fibrils formed in the presence and in the absence of eugenol. Our FTIR data, as shown in Supplementary Figure S7, shows the second derivative spectra of the aggregate samples after smoothening and baseline correction (subtracting the baseline of only eugenol sample, as shown in Supplementary Figure S10) in the amide I region (1700 cm^−1^ to 1600 cm^−1^). The signatures of all the FTIR curves look identical to each other (Supplementary Figure S7). We observed several characteristic peaks (1615 cm^−1^, 1636 cm^−1^, 1652 cm^−1^, 1670 cm^−1^ and 1684 cm^−1^) for all the obtained FTIR curves. The peak at 1615 cm^−1^ suggests the occurrence of intermolecular β-sheets and both peaks at 1670 cm^−1^ and 1684 cm^−1^ indicate the presence of β-turns. Information obtained from FTIR data on the structural properties reveals that the fibrils generated from both inhibited and uninhibited reactions have similar secondary structures. It is possible that eugenol suppresses the aggregation process without affecting the aggregation pathway.

### Isothermal Titration Calorimetry (ITC) results on eugenol-insulin interaction

**Figure 4:**
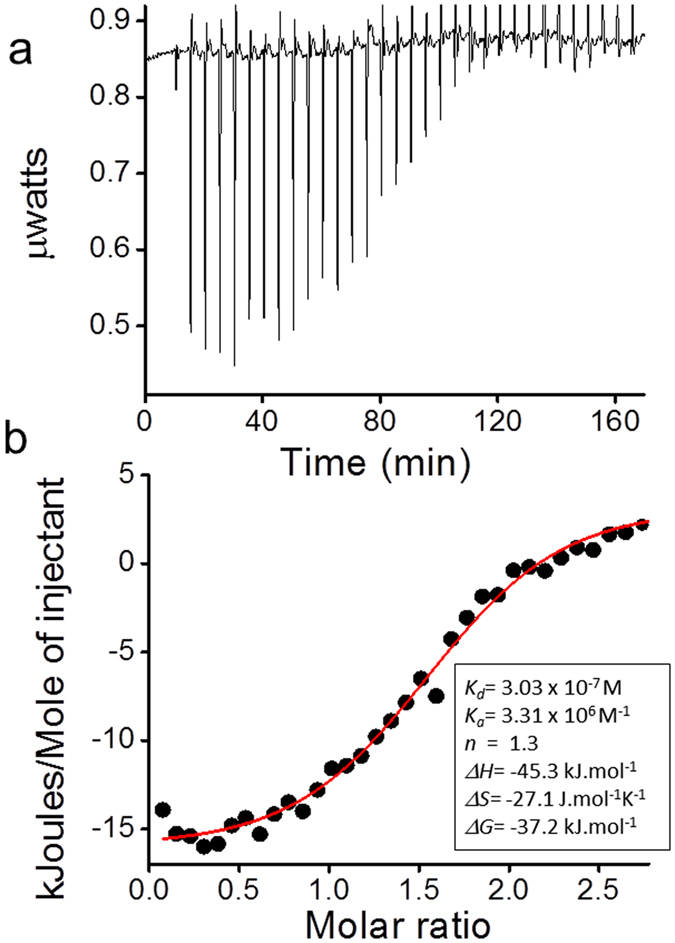
Interaction of insulin with eugenol by Isothermal Titration Calorimetry (ITC). (**a**) Raw data (after baseline subtraction) obtained from ITC for titration of insulin (10 μM) with ligand eugenol (125 μM) in PBS at pH 7.4 and at 298 K, revealing the calorimetric response peaks in μWatt during successive injections of eugenol to the insulin sample cell. Raw signal obtained from ITC is shown in Supplementary Figure S13. (**b**) Integrated heat profile of ITC data showing the binding enthalpies corrected for heat of ligand injection. The solid line represents the minimized independent fitting by a model of one site per monomer (using Nano Analyze 2.1 software from Thermal Analysis). Inset of panel *b* lists the thermodynamic parameters obtained for insulin-eugenol interaction.

ITC is considered as one of the robust method to understand biomolecular interactions and to extract important thermodynamic parameters linked to protein-ligand binding^37^,^38^. To understand interaction of eugenol with native insulin species, we carried out ITC measurements and the obtained results are shown in Fig. 4. The results clearly indicate affinity of eugenol for native insulin structure, revealing important thermodynamic parameters of eugenol-insulin interaction, as shown in the inset of Fig. 4b (*K*_*a*_ = 3.31 × 10^6^ M^−1^, *K*_*d*_ = 3.03 × 10^−7^ M, Δ*H* = −45.3 kJ.mol^−1^, Δ*S* = −27.1 J.mol^−1^ K^−1^, ΔG = −37.2 kJ.mol^−1^). The data indicate a single binding site for insulin-eugenol interaction (n = 1.3). These results correlate well with the obtained results from our quenching experiments (Supplementary Figure S5). It has been reported that the eugenol molecule binds to the odorant-binding protein (OBPs) with similar thermodynamic parameters and affinity constants^38^.

### Protective effect of eugenol on amyloid-induced hemolysis

**Figure 5:**
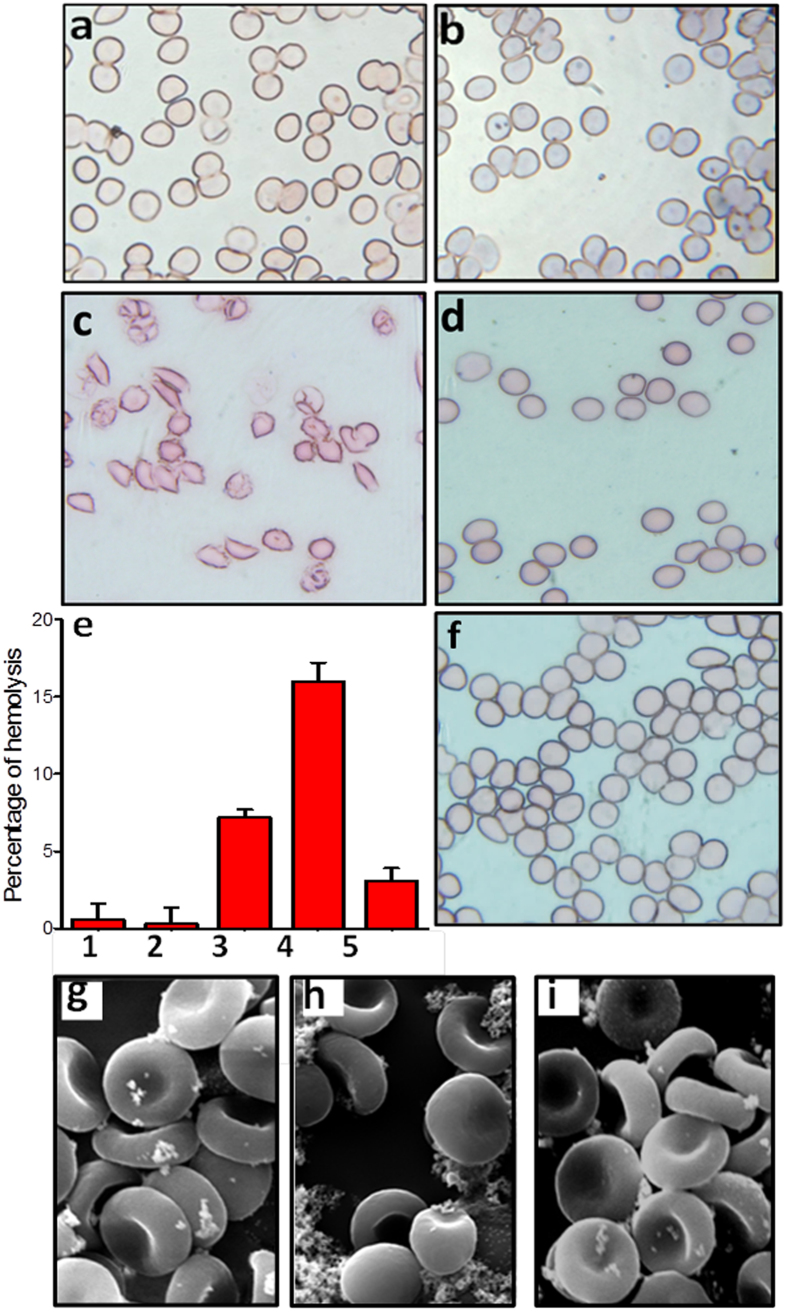
Protective effect of eugenol against amyloid induced hemolysisof RBCs in PBS buffer (pH 7.4) and at 37 °C. (**a**) Optical microscopy images of only RBCs in PBS buffer. (**b**) RBCs + 2 μM of soluble serum albumin. (**c**) RBCs + 2 μM of amyloid aggregates of serum albumin. (**d**) RBCs + 2 μM of amyloid aggregates of serum albumin + 0.9 mM eugenol. (**e**) Histogram showing percentage of hemolysis caused by amyloid fibrils of serum albumin in the presence and in the absence of eugenol: (**1**) 5 μM soluble BSA; (**2**) 0.9 mM eugenol; (**3**) 5 μM aggregated BSA (**4**) 30 μM aggregated BSA; (**5**) 30 μM aggregated BSA + 0.9 mM eugenol. (**f**) RBCs + 0.9 mM eugenol. (**g**) SEM images of control RBCs. (**h**) SEM images of lysed RBCs in the presence of BSA aggregates. (**i**) SEM images of RBCs + amyloid sample in the presence of eugenol.

Finally, we extended this investigation to explore whether eugenol has any protective effect on amyloid linked biological complications. Because the proteins chosen for the current study are known to occur in the blood stream, we decided to investigate the effect of eugenol on amyloid induced lysis of red blood cells, using BSA as a model amyloid system. Though investigations on the damaging effects of amyloids on erythrocytes are yet to begin on a large scale, Aβ amyloids are already known to cause hemolysis by damaging the red blood cells and inhibition of such hemolysis have also been reported in the presence of selected flavonoids^39^,^40^. Optical images obtained from hemolysis experiments are shown in Fig. 5. As expected, control RBCs in PBS buffer (Fig. 5a) and RBCs incubated with soluble BSA (Fig. 5b) did not show any indication of lysis. Further, we did not notice any indication of hemolysis when RBCs were treated with only eugenol (Fig. 5f). However, in the presence of BSA amyloids, we observed lysed RBCs, suggesting severe damage caused by amyloid aggregates (Fig. 5c and Supplementary Figure S11). The morphology of the lysed RBCs looks similar to the morphology observed for lysed RBCs in the presence of Aβ amyloids as reported in a recent investigation^40^. Interestingly, when we added BSA amyloid sample, which was pre-incubated with eugenol, to a suspension of RBCs, no such lysis was observed (Fig. 5d). From the absorbance spectra of the RBC samples we calculated the percentage of hemolysis and the obtained data, as shown in Fig. 5e, largely support the observed data obtained from microscopic studies. We further confirmed the inhibition of hemolysis in the presence of eugenol by SEM imaging studies and the data are shown in Fig. 5g,h,i.

## Discussion

Current study clearly indicates that eugenol has the ability to inhibit or to interfere with the amyloid aggregation of insulin and serum albumin. We also find that eugenol can prevent rapid coaggregation of [lysozyme + insulin + serum albumin] sample. One of the factors that trigger the onset of temperature induced amyloid formation is believed to be the increase in the population of aggregation prone protein intermediate (I) species^41^,^42^. Besides that these intermediate species, with exposed hydrophobic side chains, are known to promote intermolecular interactions leading to formation of amyloids. Because protein-ligand interactions play a key role in preventing protein aggregation^17^, it is possible that the occurrence of eugenol-protein interactions may stabilize the native conformations to shift the equilibrium of N ⇋ I toward the left, thus reducing the population of the aggregation-prone intermediate species. Soldi *et al*. have suggested that the stabilization of native proteins due to ligand binding is an important factor for prevention of amyloid aggregation and such inhibition effect should be independent of the aggregation pathways^17^. In addition to its affinity for protein monomers, eugenol seems to have the potential for binding to the aggregated species. Our data on the inhibition of amyloid-induced hemolysis (Fig. 5), binding of eugenol with insulin (Fig. 4) and suppression of both spontaneous and seed-induced aggregation of proteins (Fig. 1) mostly agree to the ability of eugenol to bind to both the monomeric as well as aggregated species of proteins. The interaction of eugenol with BSA has already been reported by Fujisawa *et al*.^6^. Our data obtained from both CD and ThT measurements largely agree to this mechanism of stabilization of monomers because we observed the retention of native like structures in the presence of eugenol during the conversion of native conformation to β-sheet assembled structures (Fig. 3a,b, Fig. 3c,d). Our ITC data further confirm viable interaction of eugenol with native soluble insulin molecules (Fig. 4).

Eugenol has an interesting structure in which an aromatic moiety is linked to a hydrocarbon chain, a hydroxyl group and a methoxy group. A molecule with such a unique structure is predicted to participate in multiple non-covalent interactions with globular proteins, provided that it finds an appropriate binding domain within the protein molecule. Both methoxy and hydroxyl groups are known to be vital for eugenol’s antioxidant property^43^. The presence of phenolic group in the inhibitor molecules and the aromatic amino acids in the amyloid prone sequence of the proteins is believed to facilitate the interaction between the inhibitor and the amyloidogenic core of the proteins, suppressing the fibril assembly^44^. Studies have suggested that eugenol is capable of interacting with protein domains through multiple binding poses^45^. Recent study on eugenol-albumin interaction have already shown that eugenol possess strong binding affinity for surface exposed lysine residues, facilitated by 4′-OH group of eugenol^46^.

On the issue of the protective effect of eugenol against amyloid-induced hemolysis, we predict that eugenol molecules may interact with BSA amyloids and such interaction may perhaps suppress the lysis effect on RBCs. This mechanism is supported by an earlier report by Mattson *et al*., where the suppression of Aβ-induced damaging effect on the erythrocytes has been reported in the presence of an amyloid binding dye^39^. Some studies have also revealed that eugenol suppresses the toxic effect of Aβ amyloids on PC12 cells^12^,^13^. Additionally, our data on the affinity of eugenol for amyloid aggregates as shown in Supplementary Figure S6 further support this hypothesis. The issue of what concentration of eugenol is required to prevent amyloid formation in our study, we had to add 1–6 mM of eugenol to the aggregating protein samples under *in vitro* conditions in order to see inhibition effect. However, in our hemolysis assays, inhibition of cell lysis was observed in the presence of ~0.9 mM eugenol whereas only eugenol at that concentration did not have any damaging effect on RBCs. Earlier studies involving mouse models as well as cell models have already reported the use of eugenol at concentrations in the millimolar range^14^,^47^. Further investigation would be required to clarify the issue of effective concentration of eugenol and its relevance to different biological systems.

The eugenol molecule seems to be a very promising candidate for prevention of protein amyloid formation. This study also reveals eugenol’s ability to protect erythrocytes against amyloid induced hemolysis. Stabilization of the native protein structures seems to be crucial for the inhibition of protein aggregation in the presence of eugenol. Further experiments are certainly required to confirm this inhibition effect on other amyloidogenic proteins and peptides. However, for therapeutics against medical severities associated with insulin aggregation, the design of eugenol based drugs could be beneficial.

## Methods

### Chemicals

Eugenol (from natural source with purity of ≥98.5%) and BSA were procured from Sigma-Aldrich. Insulin and lysozyme proteins were obtained either from HIMEDIA (India). All other reagents and chemicals used in this work were purchased either from HIMEDIA or Sigma-Aldrich. Extinction coefficients used for proteins were as follows: 43824 M^−1^ cm^−1^ at 280 nm for BSA and 6080 M^−1^ cm^−1^ at 278 nm for insulin. Eugenol stock solution was prepared by dissolving it in ~70% ethyl alcohol solution.

### Fluorescence studies

A Perkin Elmer LS 55 fluorescence spectrometer was used for all fluorescence related experiments in the current study. For monitoring the quenching of eugenol in the presence of insulin, we measured the fluorescence emission of the eugenol sample in the presence of different concentrations of insulin. The *λ*_ex_ and *λ*_em_ values used for eugenol were 262 nm and 325 nm respectively^5^. To obtain fluorescence emission spectra of eugenol, for data analysis, the spectra of insulin sample were subtracted from the respective spectra obtained for [insulin + eugenol] samples. To study the amyloid aggregation kinetics of protein, Thioflavin-T binding assay was used following the established protocol^25^,^48^.

### Amyloid aggregation of proteins

Amyloid aggregation was achieved by incubating the protein monomer samples in PBS at ~70 °C in the presence and absence of eugenol^19^,^22^,^49^. For conducting seed-induced amyloid aggregation reactions, preformed amyloid fibrils (~15% weight/weight) of respective proteins were used as seeds. To study co-aggregation of proteins^25^, we incubated mixed monomers of insulin, lysozyme and BSA in the presence and absence of eugenol (~3 mM). Since eugenol stock solution was prepared in ethanol, we tested the effect of ethanol (at a maximum concentration value that was used in the current study) on amyloid formation of proteins. Data are shown in Supplementary Figure S1.

### Sedimentation assay for eugenol-amyloid affinity

The sample containing eugenol and amyloid fibrils was incubated for 20 min and then centrifuged at 25200 rcf. The concentration of eugenol in the supernatant was then measured using UV-visible spectrophotometer (SHIMADZU 1800). We varied the concentrations of protein aggregates while keeping the concentration of eugenol constant for all the samples. All data were base line corrected.

### Atomic force microscopy (AFM)

Atomic force microscopy measurements were performed using XE-70 Park Systems. For AFM measurements, [insulin + eugenol] aggregate sample was diluted (10 folds) and from this diluted sample, an aliquot of 20 μL was kept on freshly cleaved mica and then it was allowed to dry at room temperature. Images were taken immediately using tapping mode (NC-AFM) with a resonance frequency of 300 Hz and a set point value of 11.3 nm. All AFM images were captured under ambient condition.

### Scanning Electron Microscopy

A Carl Zeiss EVO18 SEM was used to see the morphology of lysed RBCs. The RBC pellet was fixed using 1% glutaraldehyde and the sample was then incubated at 37 °C for 1.5 h followed by postfixation with 1% osmium tetroxide in PBS. Cells were then treated with increasing concentrations of ethanol. The cell suspensions were dropped onto silver stubs, dried, and sputtered with gold before viewing under scanning electron microscope.

### ATR-FTIR experiments

A Bruker Vertor 70 spectrometer (equipped with silicon carbide source and MCT detector) was used for obtaining FTIR spectra of mature amyloid fibrils. OPUS 6.5 software (Bruker Co., Germany) was used for data processing. All original spectra of amyloid fibrils of different proteins formed in the presence and absence of eugenol were processed for baseline correction between 1700 cm^−1^ and 1600 cm^−1^ for further analysis.

### Native Gel-electrophoresis

Native (non- denaturing) polyacrylamide gel electrophoresis was performed at a constant voltage of 25 mA with a mini-PROTEIN II Bio-Rad electrophoresis system using a Tris-HCl polyacrylamide gel. The gels were stained with Commassie Blue protein gel stain^50^. The molar ratio of BSA:eugenol was maintained at 1:450 where protein concentration was ~5 μM and the molar ratio of insulin:eugenol was maintained at 1:150 where the protein concentration was 43 μM. We loaded ~3 μg of protein samples per well. Aliquots of equal volumes were loaded in each well taken from different samples. The mature fibrils were directly loaded to the gel without any processing.

### Circular dichroism spectroscopy (CD)

We performed CD experiments by using JASCO CD spectrometer (model J-815-150 L) with attached Peltier temperature controller. The path length of the cell was 2 mm. We obtained CD spectra of samples in the presence and absence of eugenol at different time points of the aggregation reaction.

### Isothermal Titration Calorimetry (ITC)

The binding studies of eugenol molecule with native insulin were performed using NanoITC (TA Instruments, USA) at 298 K. ITC titrations of 10 μM of insulin with 125 μM eugenol were performed. All solutions were degassed under vacuum to eliminate air bubble formation inside the calorimeter cell. The reference cell was filled with double-distilled and degassed water. During titration 1 μl of the eugenol was injected to insulin containing sample cell at an interval of 300 s. To correct the heat effects of dilution and mixing, control experiments were performed at the same concentrations of the insulin and eugenol and subtracted from the respective insulin-eugenol titrations. The data were analyzed and best-fitted using Nano Analyze 3.6 software to extract the thermodynamic parameters of the protein-ligand interaction.

### Hemolysis assay

The blood sample was collected from a healthy volunteer donor and all methods were carried out in accordance with relevant guidelines and regulations of Ethics Committee of Indian Institute of Technology Jodhpur, India. All the experimental protocols were approved by Ethics Committee of Indian Institute of technology Jodhpur (Approval letter no IITJ/EC/2016/02-D). Informed consent was obtained from all the subjects involved in this study prior to conducting experiments. Established protocol^39^ was followed to study the effect of eugenol on amyloid-induced lysis of Red Blood Cells (RBCs). RBCs were separated by centrifuging the blood sample at 2138 rcf for 10 min. The pellets of RBCs were collected and washed thrice with sterile phosphate buffer saline (PBS). The pellet was then re-suspended in PBS to make a diluted (~3 times) stock solution, from which an aliquot of ~100 μl was added to different protein samples for conducting the lysis assays. Concentrations of the BSA monomers and BSA aggregates were maintained at ~5 μM and a higher concentration of ~30 μM. Suppression of BSA-amyloid induced hemolysis in the presence of eugenol was carried out at ~0.9 mM of the inhibitor. Control experiments were performed to see the effect of only eugenol. After addition of the samples (BSA, BSA aggregates, eugenol, BSA aggregates + eugenol, PBS as negative control, and water as positive control) these suspensions were slightly vortexed and incubated in static condition for four hours at 37 °C. The samples were then vortexed and centrifuged at 2138 rcf for 10 min. The obtained pellet was then smeared, stained (Leishman’s stain) and visualized under optical microscope (at 100x magnification). The absorbance spectrum of the supernatant obtained from each sample, after centrifugation step, was recorded using UV-visible spectrophotometer (UV 1800 Shimadzu). The percentage hemolysis was calculated based on the absorbance (A) at ~576 nm. The percentage lysis was calculated by following the formula given below.


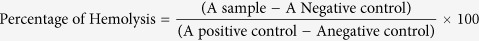


### Data-processing

All plots and graphs displayed in this article were obtained by using Origin-2015 graphics and analysis tool. Linear fit analysis for calculating Stern-Volmer constant and associated binding constant parameter was also performed using Origin-2015 software. Data obtained from ITC experiments were processed and analyzed by Nano Analyze v3.6 software from Thermal Analysis.

## Additional Information

**How to cite this article**: Dubey, K. *et al*. Eugenol prevents amyloid formation of proteins and inhibits amyloid-induced hemolysis. *Sci. Rep.* **7**, 40744; doi: 10.1038/srep40744 (2017).

**Publisher's note:** Springer Nature remains neutral with regard to jurisdictional claims in published maps and institutional affiliations.

## Supplementary Material

Supplementary Information
