# Supplementary material for: Eugenol prevents amyloid formation of proteins and inhibits amyloid-induced hemolysis
Source: Sci Rep. 2017 Feb 1;7:40744. doi: 10.1038/srep40744 (PMC5286398; doi:10.1038/srep40744)
Supplement: Supplementary Information [file srep40744-s1.pdf]

Eugenol prevents amyloid formation of proteins and inhibits amyloid-induced hemolysis.

Kriti Dubey[b], Bibin G. Anand[b], Dolat Singh Shekhawat[b], and Karunakar Kar[a]*

[a] School of Life Sciences, Jawaharlal Nehru University, New Delhi-110067; [b]Department of Biology, Indian Institute of Technology Jodhpur.

Supplementary Information

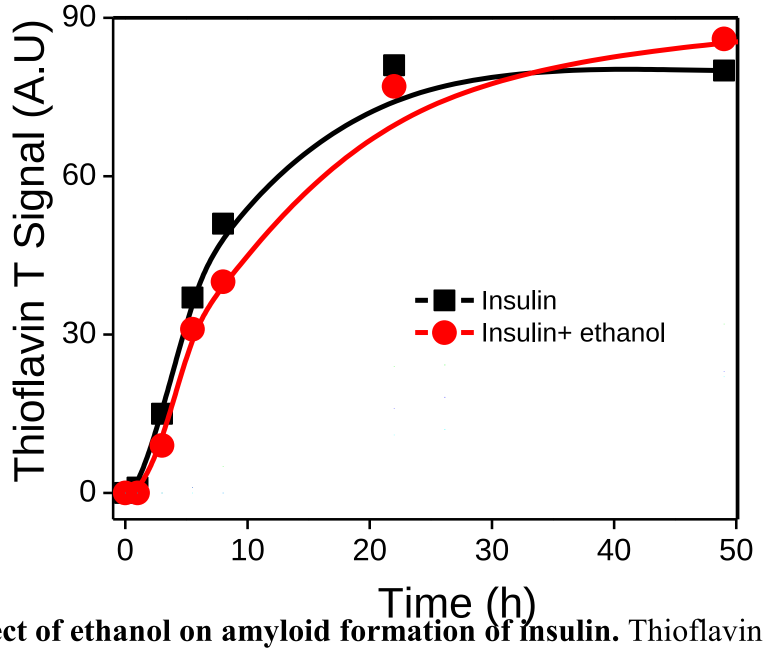

Figure S1. Effect of ethanol on amyloid formation of insulin. Thioflavin T reading of insulin sample in the presence (●) and absence (■) of ethanol. Since eugenol stock solution was prepared in ~70% ethanol solution, we added 15µL of 70% ethanol (is equivalent to maximum volume of eugenol aliquot used in this study) to insulin sample. This experiment was done to confirm that the inhibition effect of eugenol is not due to the presence of ethanol in the sample.

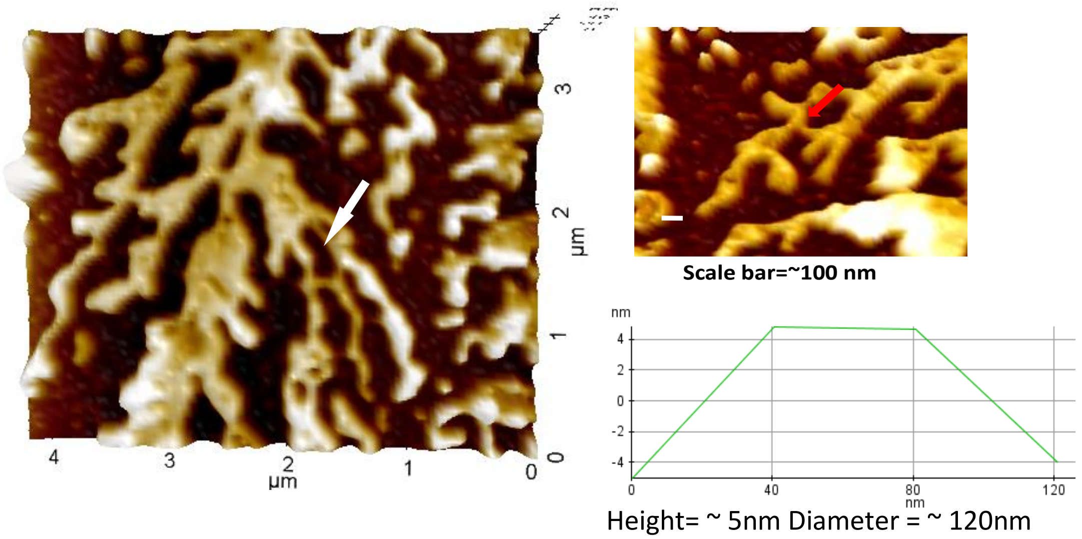

Figure S2. AFM images of mature amyloid fibrils of insulin in the absence of eugenol.

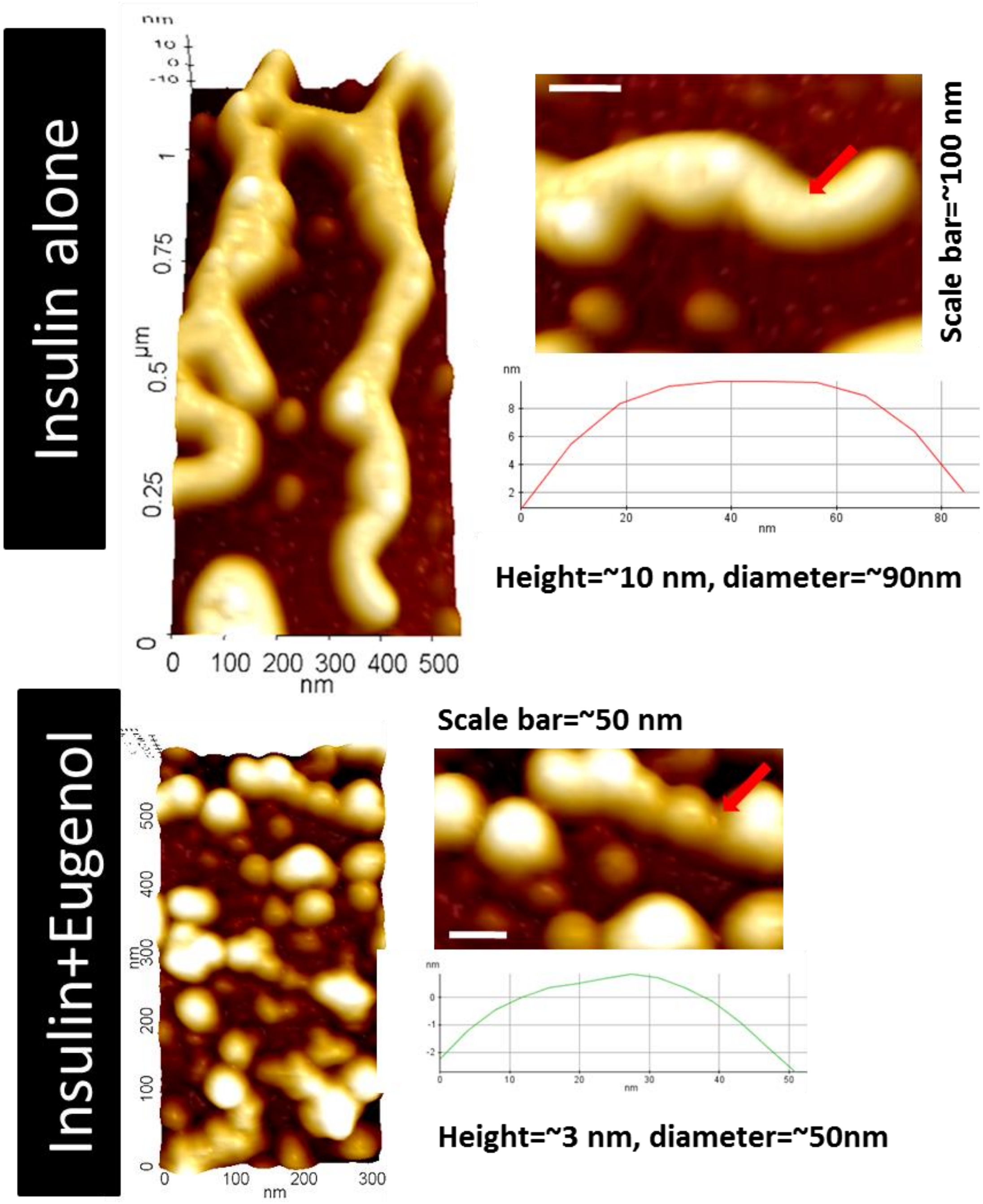

Figure S3. **Comparison of structural properties of mature fibrils of insulin obtained from eugenol inhibited reaction with the fibrils obtained from an uninhibited reaction at 5h time point.** Panel **(a)** represents mature amyloid fibrils of insulin from an uninhibited aggregation reaction. **(b)** spheroidal oliogomers obtained from an inhibited aggregation reaction of insulin in the presence of eugenol.

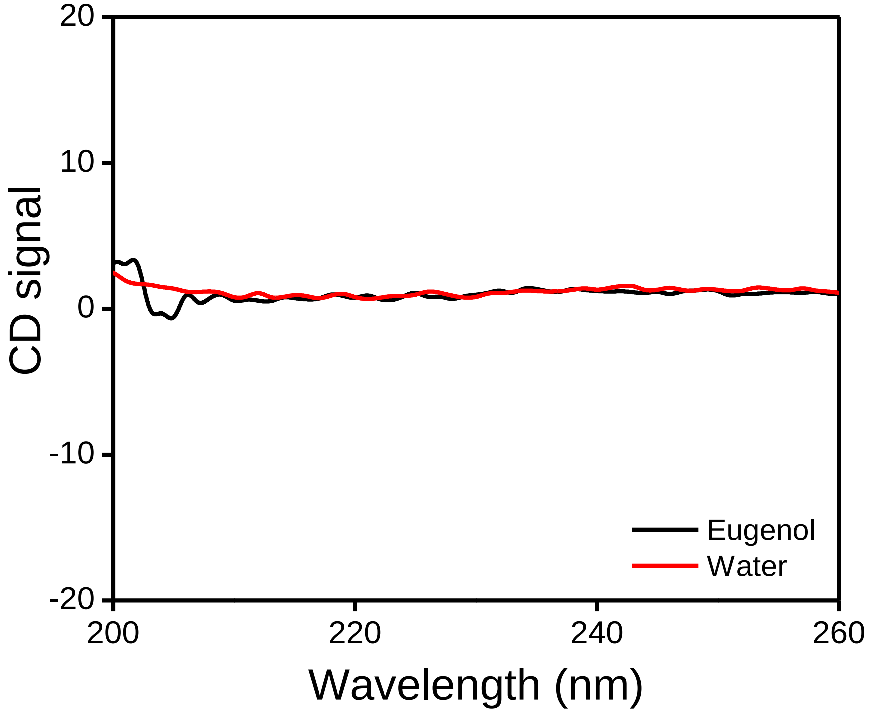

Figure S4. CD spectra of eugenol at~ 0.4 mM (black curve) and water (red curve). This experiment confirms that, at the studied concentration, eugenol does not have any CD signal.

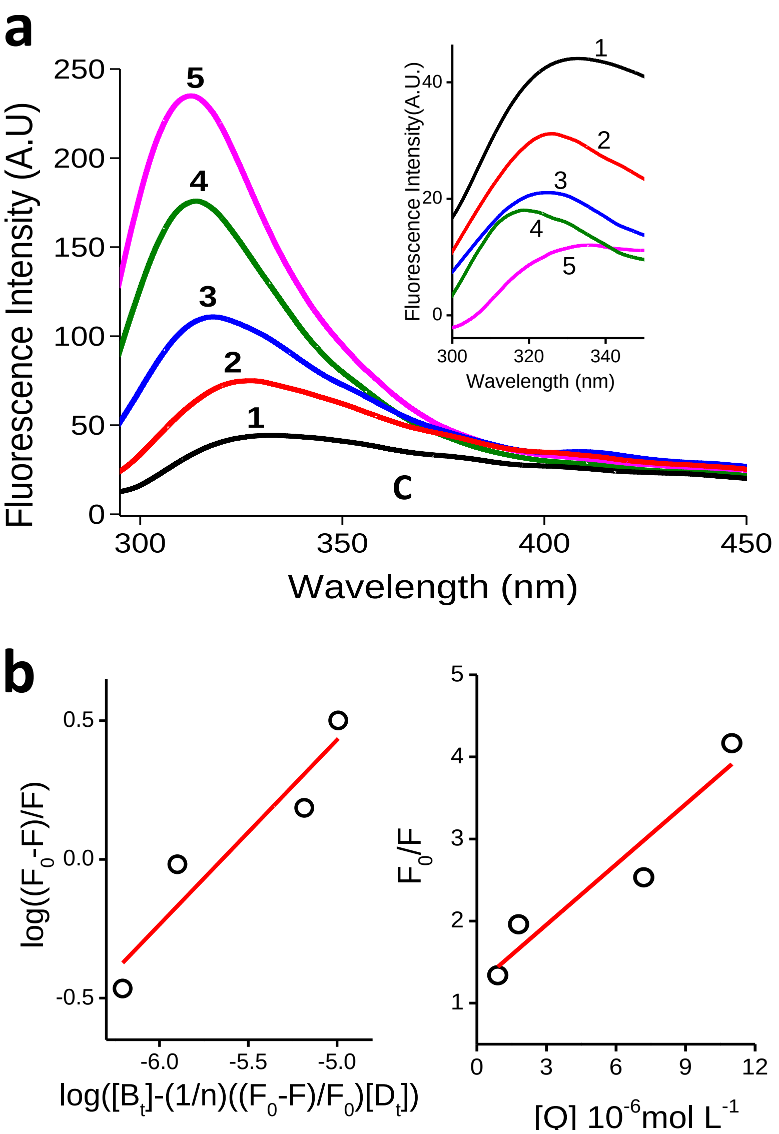

Figure S5. (a) Fluorescence spectra of eugenol sample (at 1.65 µM) with increasing concentrations of insulin. Excitation wavelength was 262 nm. Concentrations of insulin were: 1) 0 µM; 2) 1 µM; 3) 2 µM; 4) 7 µM; 5) 11 µM. The inset shows the baseline corrected emission curves of eugenol. (b) The curve of log $((F_0\text{-}F)/F)$ versus log $([B_t]\text{-}(1/n)[D_t]\ (F_0\text{-}F)/F_0)$ for eugenol binding with insulin. (c) Stern-Volmer plot of eugenol and insulin. All the fluorescence measurements were carried out at room temperature.

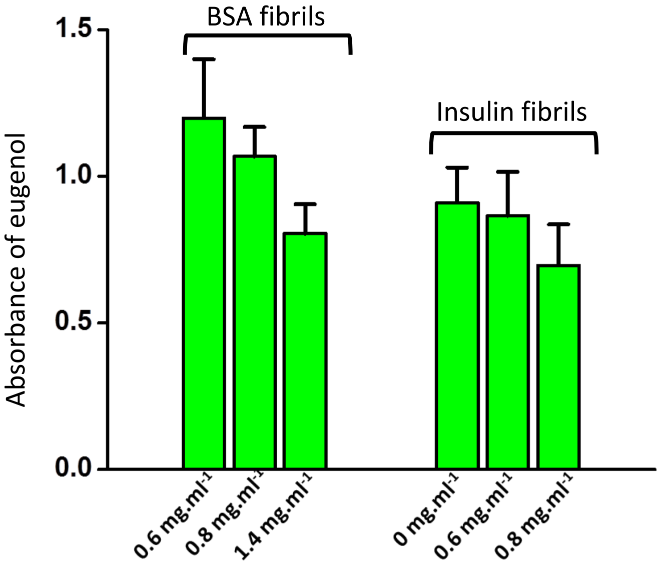

Figure S6. Sedimentation assay for determining the affinity of eugenol for amyloid fibrils of proteins. Set one in the left panel shows the decrease in the concentration of eugenol in the sample with increasing concentrations of mature amyloid fibrils of BSA. Set two in the right panel shows the decrease in the concentration of eugenol with increasing concentration of mature amyloids of insulin. Absorbance of eugenol was monitored at 280 nm. All the data are base line subtracted to make sure that the signals are due to the presence of eugenol only.

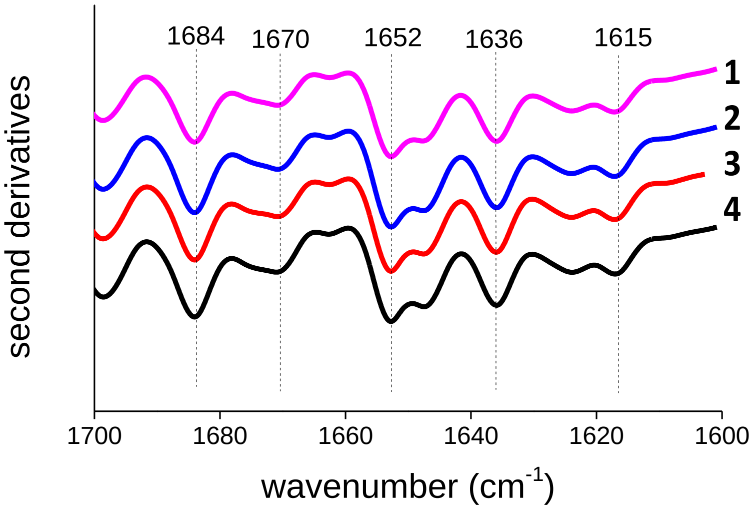

Figure S7. ATR FTIR second derivative spectra of final aggregates: 1) insulin aggregates in eugenol (molar ratio of protein: ligand is 1:100); 2) insulin aggregates in the absence of eugenol; 3) BSA aggregates in eugenol (molar ratio of protein: ligand is 1:400); 4) BSA aggregates in the absence of eugenol.

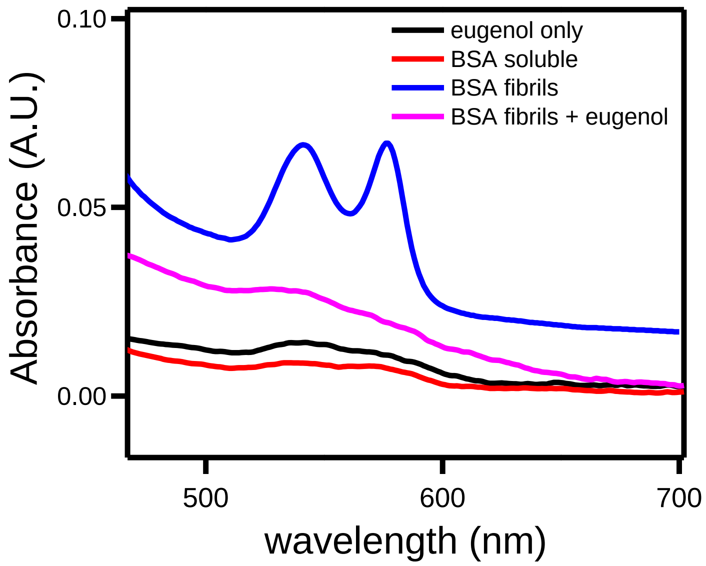

Figure S8. Absorption spectra obtained for the hemolysis experiment after four hours of incubation at 37°C. Lysis was observed in the presence of 30μM BSA amyloid fibrils (blue curve). Inhibition of BSA-amyloid induced lysis in the presence of 0.9 mM eugenol (magenta curve). Effect of soluble BSA (red curve) and only eugenol (black curve).

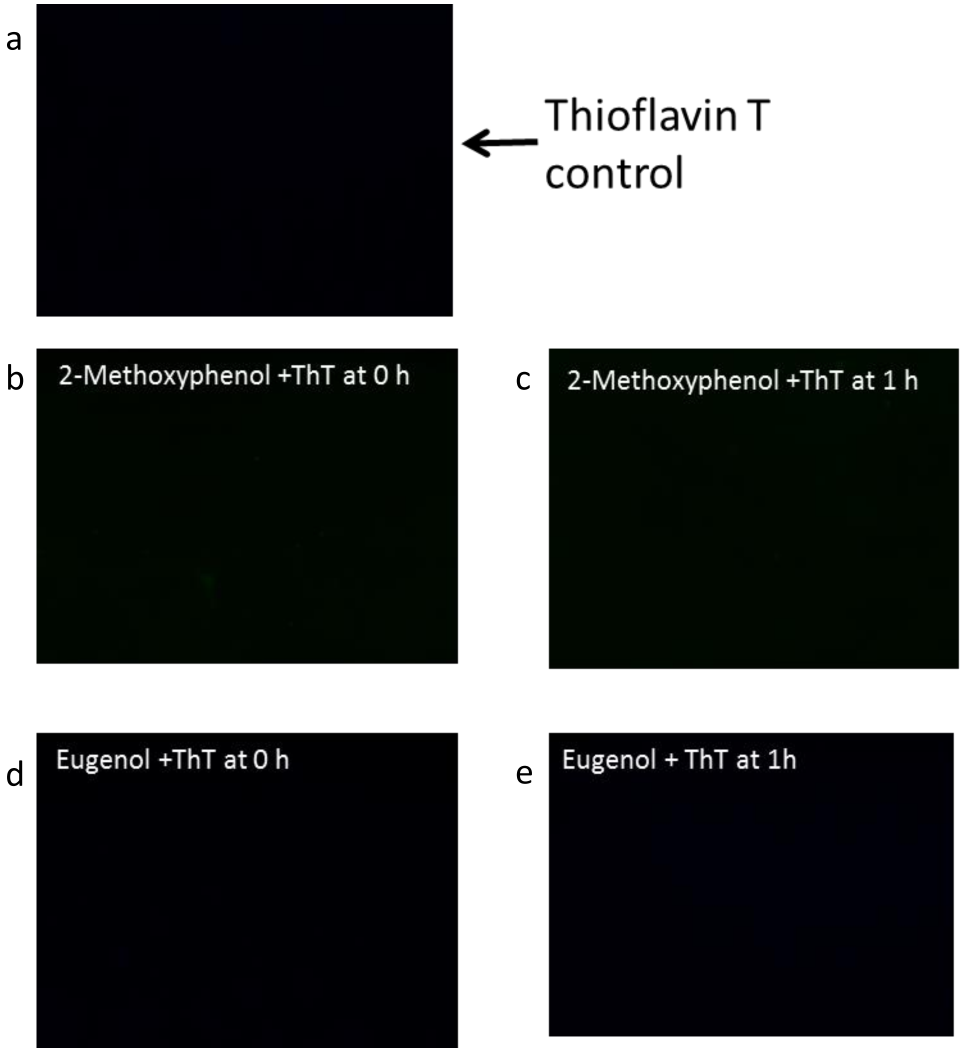

Figure S9. **Fluorescence microscopy images of Thioflavin T stained samples.** (a) Control ThT; (b) ThioflavinT + Methoxyphenol at 0h; (c) ThioflavinT + Methoxyphenol at 1h; (d) ThioflavinT + eugenol at 0h; (e) ThioflavinT + eugenol at 1h

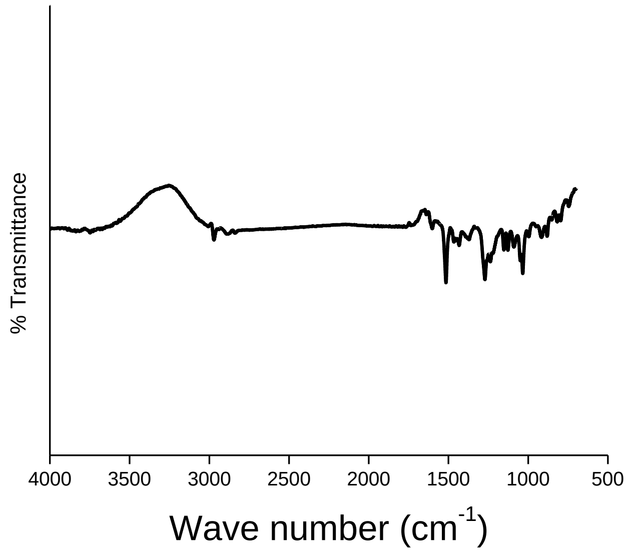

Figure S10. ATR FTIR second derivative spectra of eugenol only.

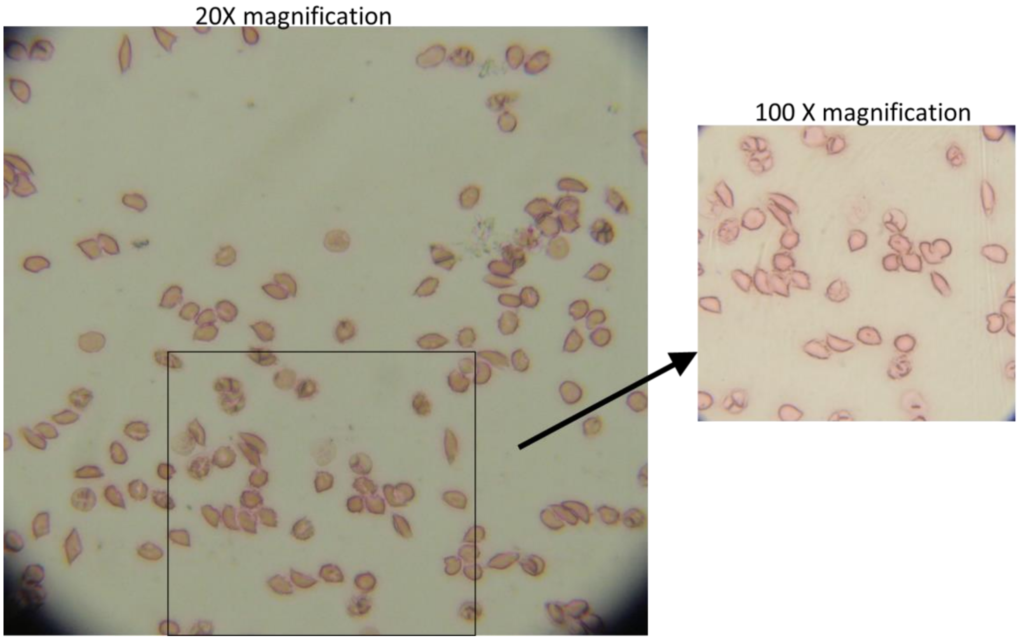

Figure S11. Microscopic images of lysed RBCs in the presence of BSA aggregates at different magnification.

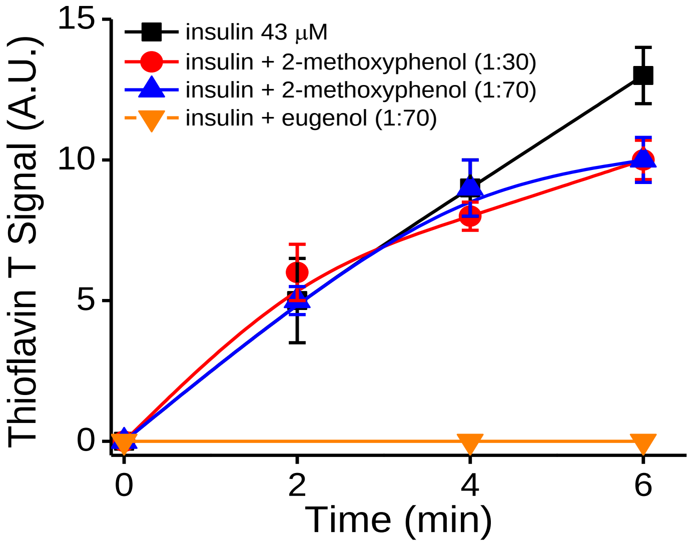

Figure S12. Comparison of the effect of 2-methoxyphenol and eugenol on amyloid formation of insulin. (■) control insulin at 43 μM; (▼) insulin + eugenol (at 1:70 molar ratio); (●) insulin + 2-methoxyphenol (at 1:35 molar ratio); (▲) insulin + 2-methoxyphenol (at 1:70 molar ratio).

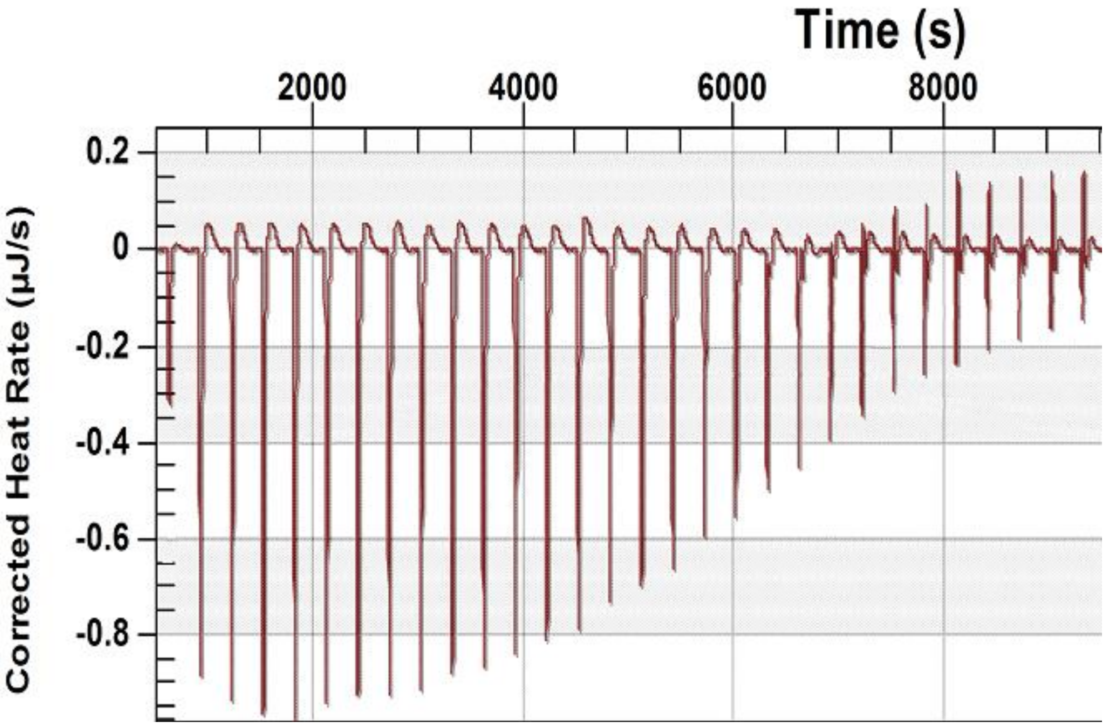

Figure S13. Raw data of ITC showing snapshot image from the instrument showing titration of insulin with eugenol.

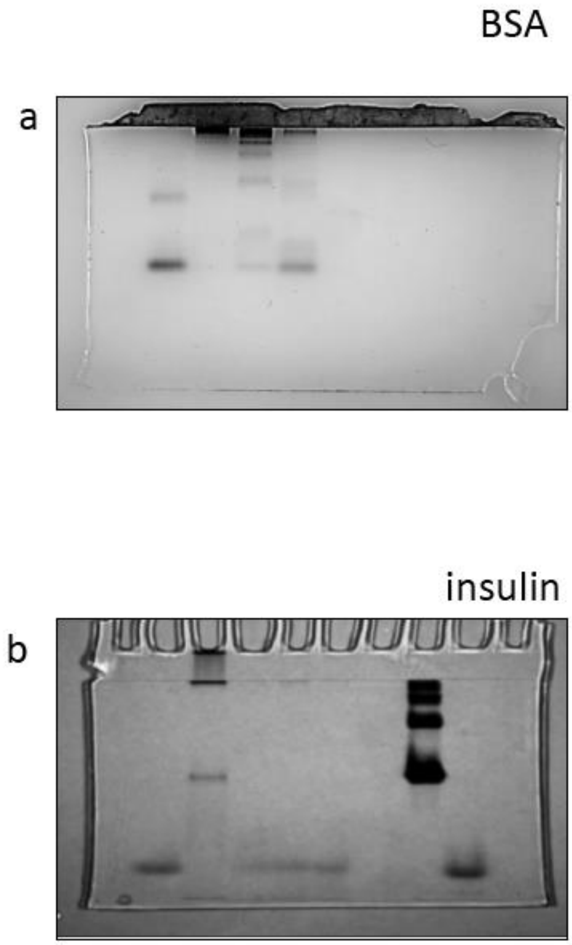

Figure S14. The uncropped native gel data obtained for both BSA(a) and insulin(b) . The cropped images are shown in panel e and panel f of *figure 3* of the main text.

Additional information on the Quenching Experiments

Natural life time of eugenol is ~10^{-8} sec. In this study, eugenol is a fluorophore and insulin is a quencher. Eugenol concentration was kept constant, i.e. ~1.65 µM whereas insulin concentrations were varied as: 1 µM, 2 µM, 7 µM, and 11 µM.

The fluorescence emission spectra of eugenol was analyzed using Stern-Volmer equation [1-4]

$$\frac{Fo}{F} = 1 + K_q \tau_{\circ}[Q] = 1 + K_{sv}[Q]$$

Where, F_0 and F are the fluorescence intensities in the absence and presence of the quencher, respectively. [Q] is the quencher concentration, K_q is the biomolecule quenching rate constant, τ_0 is the average lifetime of molecules in the absence of quencher and its value is ~ 10^{-8} s, and K_{sv} is Stern-Volmer quenching constant. The maximum dynamic rate constant of various quenchers with the biopolymer is 2.0×10^{10} L mol^{-1} s^{-1} [1]. The Stern-Volmer plot was plotted against F_0/F vs [Q], the values of K_{sv} and K_q were 2.445×10^5 L mol^{-1} and 2.445×10^{13} L mol^{-1} s^{-1} respectively. It is observed that the value of K_q is greater than 2.0×10^{10} L mol^{-1} s^{-1}, which indicates that the quenching process of eugenol by insulin was static.

Binding constant and binding site

Since the fluorescence quenching of the eugenol by insulin was static, the binding constant (K_a) and binding site (n) can be calculated by following relation [1]:

$$log\frac{F_0 - F}{F} = logK_a + \frac{1}{n}\, log\left([B_t] - \frac{1}{n}\frac{F_0 - F}{F_0}[D_t]\right)$$

[D_t] is the total eugenol concentration and [B_t] is the total insulin concentration. On the assumption that 1/n in the bracket was equal to 1, the curve $\log\frac{F_0-F}{F}$ versus $\log\left([B_t] - \frac{1}{n}\frac{F_0-F}{F_0}[D_t]\right)$ was drawn and fitted linearly. So the slope of 1/n could be obtained. If the obtained slope was not equal to 1/n, then it was substituted into the bracket and the

curve $\log\frac{F_0-F}{F}$ versus $\log\left([B_t]-\frac{1}{n}\frac{F_0-F}{F_0}[D_t]\right)$ was drawn again [1]. This process was repeated till the same value of 1/n was not obtained. The final curve was plotted. The obtained binding constant (K_a) is 5.75043 L mol^{-1} and the number of binding sites (n) are 1.5, i.e. ~1.

Reference

1. Bi, S., Yan, L., Wang, Y., Pang, B., and Wang, T. (2012) Spectroscopic study on the interaction of eugenol with salmon sperm DNA in vitro, *Journal of Luminescence 132*, 2355-2360.

2. Lakowicz, J.R., and Weber, G. (1973) Quenching of fluorescence by oxygen: Probe for structural fluctuations in macromolecules, *Biochemistry 12*, 4161-4170.

3. Lakowicz, J.R. (1999) Principles of Fluorescence Spectroscopy, *Plenum Press, New York, 2nd ed*, 237.

4. Sun, Y., Zhang, H., Bi, S., Zhou, X., Wang, L., Yan, Y. (2011) Studies on the arctiin and its interaction with DNA by spectral methods, *Journal of Luminescence 131,* 2299-2306.
